# Effectiveness and Safety of Respiratory Syncytial Virus Vaccine for US Adults Aged 60 Years or Older

**DOI:** 10.1001/jamanetworkopen.2025.8322

**Published:** 2025-05-09

**Authors:** Sarah E. Fry, Pauline Terebuh, David C. Kaelber, Rong Xu, Pamela B. Davis

**Affiliations:** 1 Center for Artificial Intelligence in Drug Discovery, Case Western Reserve University School of Medicine, Cleveland, Ohio; 2 Department of Population and Quantitative Health Sciences, Case Western Reserve University, Cleveland, Ohio; 3 The Center for Clinical Informatics Research and Education, MetroHealth System, Cleveland, Ohio; 4 Department of Medicine, Case Western Reserve University, Cleveland, Ohio; 5 Department of Pediatrics, Case Western Reserve University, Cleveland, Ohio; 6 Department of Population and Quantitative Health Sciences, Case Western Reserve University, Cleveland, Ohio; 7 Center for Community Health Integration, Case Western Reserve University, Cleveland, Ohio

## Abstract

**Importance:**

Respiratory syncytial virus (RSV) is associated with hospitalization and death among older adults. Characterizing the safety and effectiveness of recently introduced vaccines against RSV is critical.

**Objective:**

To assess the safety and effectiveness of vaccines against RSV and the major adverse events among patients aged 60 years or older during the 2023-2024 RSV season.

**Design, Setting, and Participants:**

In this study using a data platform containing electronic health records for more than 270 million patients across the US, a test-negative case-control design was used to estimate vaccine effectiveness (VE), and a self-controlled case series of vaccine recipients was included to estimate vaccine-associated adverse events. Records from participants aged 60 years or older with acute respiratory infection (ARI) and testing for RSV between October 1, 2023, and April 30, 2024, were included in the VE study. For vaccine safety analysis, all participants aged 60 years or older who received the RSV vaccine from July 1, 2023, to June 30, 2024 were included. Data were analyzed from August 2024 to March 2025.

**Main Outcomes and Measures:**

Cases were those patients who tested positive for RSV, and controls were those who tested negative for RSV. Patients were classified as vaccinated if the vaccine was received at least 14 days before testing. VE against RSV-associated ARI diagnosis, emergency department or urgent care visits, or hospitalizations was estimated using (1 − odds ratio) × 100%. Excess risks of immune thrombocytopenic purpura and Guillain-Barré syndrome diagnosis for 6 weeks after vaccine administration were calculated.

**Results:**

Of 787 822 patients tested for RSV, 53 963 were positive (733 859 were controls); 1318 cases (2.4%) and 66 928 controls (9.1%) were vaccinated. Overall, VE was 75.1% (95% CI, 73.6%-76.4%) against ARI and was similar for age groups of 60 to 74 years and 75 years or older and against urgent care visits or hospitalizations. Immunocompromised patients had a VE from 67.0% (95% CI, 62.6%-70.9%) for patients aged 60 to 74 years to 73.1% (95% CI, 69.9%-76.0%) for those aged 75 years or older, and the lowest VE (ie, from 29.4% [95% CI, 3.5%-48.4%] for patients aged 60-74 years to 44.4% [95% CI, 1.0%-68.8%] for those aged ≥75 years) was for a subgroup of patients who received stem cell transplants. Among 4 746 518 vaccine recipients, no excess risk of immune thrombocytopenic purpura diagnosis was detected. An excess of 5.2 cases (RSVPreF3+AS01) or 18.2 cases (RSVPreF) of Guillain-Barré syndrome were diagnosed per 1 000 000 doses of RSV vaccine administered.

**Conclusions and Relevance:**

VE for the RSV protein subunit vaccine in this case-control study was similar to the VE in clinical trials. The VE for immunocompromised patients was mildly (overall) to moderately (for stem cell transplant recipients) diminished. Risk of immune thrombocytopenic purpura after vaccination was not elevated, but the risk of Guilain-Barré syndrome was statistically significantly elevated in patients who received the RSVPreF vaccine but not in those who received RSVPreF+AS01 vaccine, although the risk was small. These observations should inform clinicians’ choices and patient instructions.

## Key Points

**Question:**

What is the effectiveness of vaccines against respiratory syncytial virus (RSV) among older adults and at-risk subgroups, and are there major adverse events associated with vaccination?

**Findings:**

In this case-control study of 787 822 patients tested for RSV, vaccine effectiveness was approximately 75% among adults aged 60 years or older against RSV-associated acute respiratory infection, urgent care or emergency visits, or hospitalization; effectiveness was less but still substantial among immunocompromised patients. An estimated excess of 11.2 cases of Guillain-Barré syndrome followed administration per 1 000 000 doses of RSV vaccine.

**Meaning:**

These findings suggest that RSV vaccination of older patients is effective even for most immunocompromised patients, but diagnoses of Guillain-Barré syndrome are increased after vaccination.

## Teaser

This case-control study assesses the safety and effectiveness of vaccines against respiratory syncytial virus (RSV) and the major adverse events among patients aged 60 years or older during the 2023-2024 RSV season.

## Introduction

In 2023, 2 protein subunit vaccines for respiratory syncytial virus (RSV) in adults were approved by the US Food and Drug Administration.^1,2^ Although they were initially recommended for adults aged 60 years or older using shared clinical decision-making,^3^ subsequent considerations prompted the Advisory Committee on Immunization Practices to streamline recommendations to those aged 60 to 74 years at increased risk of severe disease and all those aged 75 years or older for the upcoming 2024-2025 season.^4^ A report by Britton et al^4^ in the *Mortality and Morbidity Weekly Report* updating the recommendations from the Advisory Committee on Immunization Practices identified deficiencies in the currently available data, including the vaccine effectiveness (VE) for specific immunocompromised groups and more definitive accounting of major adverse events, such as Guillain-Barré syndrome (GBS) or immune thrombocytopenic purpura (ITP). The authors noted that updated RSV vaccine safety analyses among adults aged 60 years or older, including results from the full 2023-2024 RSV season, are needed to confirm these recommendations.

Electronic health record (EHR) data platforms are becoming increasingly large, so we used Epic Cosmos (Epic Systems), which contains more than 270 million deduplicated patient EHR records in a deidentified aggregated data platform,^5^ to estimate VE of the 2 RSV vaccines available during the 2023-2024 respiratory virus season among patients aged 60 years or older with medically attended acute respiratory illness (ARI) who were tested for RSV. In addition, VE estimates for subgroups of patients who were immunocompromised and estimation of rare major adverse events after vaccination were possible because of the large population included in Cosmos. Cosmos has been used previously to estimate VE for mpox vaccine^6^ but not, to our knowledge, to assess vaccines for more common diseases. Its large size, with updated clinical data available within several weeks of medical encounters, make it an attractive adjunct to current methods for monitoring vaccine safety and effectiveness.

## Methods

This study used deduplicated, deidentified aggregated EHR data from Cosmos, representing more than 270 million patient records from more than 1568 hospitals and 35 500 clinics from all 50 states using the Epic EHR.^5^ The MetroHealth Institutional Review Board has designated the use of deidentified, aggregate data as are found in Cosmos as not involving human participants; therefore, this study was exempt from review as per the standard stated in Section §164.514(a) of the Health Insurance Portability and Accountability Act Privacy Rule. Study design and result reporting followed the Strengthening the Reporting of Observational Studies in Epidemiology (STROBE) reporting guideline.

The methods and results assessing the distribution of RSV cases by week and by state and the percentage of positive tests for RSV using data from Cosmos are shown in the eMethods in Supplement 1. A retrospective test-negative case-control study design was used to estimate RSV protein subunit VE against RSV illness among adults aged 60 years or older with ARI-associated medical encounters, emergency department or urgent care encounters, or hospitalizations during the 2023-2024 RSV season. Eligible encounters occurred between October 1, 2023, and April 30, 2024. ARI cases were defined using *International Statistical Classification of Diseases and Related Health Problems, Tenth Revision* (*ICD-10*) encounter diagnosis codes (J00J06, J09-J18, J20-J22, and R05). Eligible patients had testing for RSV within 10 days before or 3 days after the ARI-associated encounter. RSV case patients were those who tested positive for RSV based on clinical viral testing (including reverse transcription–polymerase chain reaction tests, nucleic acid amplification tests, and antigen tests), whereas controls were those who tested negative, excluding patients with a positive RSV test result during the study period. We further evaluated VE among specific subpopulations, including among immunocompromised individuals and across transplant recipients, considering both solid organ transplants and hematopoietic stem cell transplants, as well as examining these groups separately.

Race and ethnicity were self-reported and then collapsed by Epic Cosmos into the 5 categories recognized by the Office of Management and Budget: American Indian or Alaska Native, Asian, Black, Native American or Pacific Islander, or White. Those of multiracial or other ancestry were classified as “other.” The racial and ethnic distribution for control participants was comparable. Data on race and ethnicity were included because there are known differences in racial and ethnic propensity to obtain vaccines when they are first released.

Cosmos ascertains vaccination status through records of administered vaccinations and patient-reported historical vaccinations in the contributing EHRs, as well as through external immunization data, such as state registries, when the contributing EHR is connected to these through health information exchanges. Patients were classified as vaccinated if they had any record of receiving a single dose of an RSV protein subunit vaccine from any manufacturer at least 14 days before testing for RSV and were classified as unvaccinated if they did not. Patients who received the RSV vaccine less than 14 days before RSV testing were excluded.

Risk of GBS and ITP was evaluated using a self-controlled case series design.^8^ We included participants aged 60 years or older who received the RSV vaccine between July 1, 2023, and June 30, 2024, allowing for 90 days of follow-up for all patients. GBS was defined as a billed final diagnosis of GBS (*ICD-10* code G61.0) for an inpatient visit, whereas ITP was defined using any encounter diagnosis (*ICD-10* code D69.3). A 1-year washout period was used to define incident cases of GBS and ITP. Incidence rates of GBS and ITP were compared between days 1 to 42 after vaccination (risk period) and days 43 to 90 (control period). Outcome risk was estimated using an incidence rate ratio (IRR), the ratio of the incidence rate in the risk period to the incidence rate in the control period. An IRR of greater than 1 indicates an increased risk of the outcome after vaccination. The 95% CIs were calculated using the standard error of the natural logarithm of the IRR. The number of attributable cases per 1 000 000 vaccinations was calculated using the following formula: attributable cases per 1 000 000 = {[(IRR − 1)/IRR] × number of events during risk period}/number of eligible vaccinations ×1 000 000.

### Statistical Analysis

Patient characteristics, including age, race and ethnicity, immunocompromised status, and presence of chronic lung and cardiovascular disease, were described among cases and controls using frequencies and proportions for binary or categorical variables and median (IQR) values for continuous variables (eTable 6 in Supplement 1). VE was estimated by comparing the odds of RSV vaccination among patients who were RSV positive vs RSV negative, calculated as VE = (1 – odds ratio [OR]) × 100%.^7^ The unadjusted OR was supplemented by stratified analyses by age, immunocompromised status, month of RSV test, and state of residence to evaluate potential confounders. The 95% CIs for VE estimates were calculated using the standard error of the natural logarithm of the OR. All analyses were performed using GraphPad Prism, version 10.2.3 for Mac (GraphPad Software, Inc) from August 2024 to March 2025. The *ICD-10*, Healthcare Common Procedure Coding System, and *Current Procedural Terminology* codes used to define patients who received various transplant procedures are reported in eTable 1 in Supplement 1. Except when otherwise specified, 2-sided *t* tests were used.

## Results

### RSV Infections Characterized

eFigure 1 in Supplement 1 shows the temporal pattern of RSV-associated hospitalizations and compares this pattern to that reported from the Centers for Disease Control and Prevention Respiratory Syncytial Virus Hospitalization Surveillance Network, and eFigure 2 in Supplement 1 shows the proportion of RSV tests that were positive at these time points for all those recorded in Cosmos and for patients aged 60 years or older compared with the corresponding data in the National Respiratory and Enteric Virus Surveillance System.

#### Participant Characteristics

Among the total sample size of 787 822, 53 963 cases and 733 859 controls were included in the VE analysis (Table 1) drawn from the large Cosmos population that reflects the demographics reported in the US Census.^5^ Of these, 81.6% (642 862 of 787 822) had had queries of the state vaccine system during the test period. The median age of the cases was 74 years (IQR, 67-82 years) and also 73 years (IQR, 67-81 years) for the controls. The age distribution among cases showed that 51.7% (27 923 of 53 963) were aged 60 to 74 years, whereas 48.3% (26 040 of 53 963) were aged 75 years or older. The racial and ethnic distribution was predominantly White (n = 44 929 [83.3%]), then Black (n = 6257 [11.6%]), Asian (n = 1326 [2.5%]), American Indian or Alaska Native (n = 440 [0.8%]), and Native Hawaiian or Pacific Islander (n = 153 [0.3%]). Other races and ethnicities comprised 7.6% (n = 4087) of the cases.

**Table 1.** Characteristics of Case Participants and Control Participants for Vaccine Effectiveness Analysis

Characteristic	Cases (n = 53 963)	Controls (n = 733 859)
Age, median (IQR), y	74 (67-82)	73 (67-81)
Age group, No. (%), y		
60-74	27 923 (51.7)	400 012 (54.5)
≥75	26 040 (48.3)	333 847 (45.5)
Race and ethnicity, No. (%)^a^		
White	44 929 (83.3)	590 499 (80.5)
Black	6257 (11.6)	104 971 (14.3)
Asian	1326 (2.5)	17 700 (2.4)
American Indian or Alaska Native	440 (0.8)	6306 (0.9)
Native Hawaiian or Pacific Islander	153 (0.3)	2178 (0.3)
Other^b^	4087 (7.6)	57 349 (7.8)
Immunocompromised, No. (%)	16 744 (31.0)	234 799 (32.0)
Chronic lung disease, No. (%)	16 691 (30.9)	223 065 (30.4)
Cardiovascular disease, No. (%)	39 013 (72.3)	525 406 (71.6)

^a^ The choice of race and ethnicity allowed for more than 1 selection.

^b^ Includes individuals whose race and ethnicity designation was not included in the 1997 Office of Management and Budget minimum categories.

Notably, 31.1% (n = 16 744) of the cases were immunocompromised, 30.9% (n = 16 691) had chronic lung disease, and 72.3% (n = 39 013) had cardiovascular disease. The prevalence of these conditions among controls did not differ substantially from cases in these parameters, varying less than 1 percentage point, and no adjustment was made for these comorbidities. For the vaccine safety analysis, 4 746 518 RSV-vaccinated adults without an *ICD-10* encounter diagnosis of GBS within 12 months before RSV vaccination and 4 740 401 RSV-vaccinated adults without an *ICD-10* encounter diagnosis of ITP within 12 months before RSV vaccination were included (Table 2). The median age of vaccine recipients was 74 years (IQR, 69-79 years). Baseline characteristics of vaccine recipients were similar for each manufacturer (Table 2).

**Table 2.** Characteristics of Vaccine Recipients for Analysis of Adverse Events

Characteristic	Total (n = 4 746 518)	RSVPreF3 (n = 3 070 888)	RSVPreF (n = 1 643 827)	Unspecified (n = 69 673)
Age, median (IQR), y	74 (69-79)	74 (69-79)	74 (69-80)	74 (69-79)
Age group, No. (%), y				
60-74	2 491 900 (52.5)	1 621 385 (52.8)	852 704 (51.9)	36 275 (52.1)
≥75	2 254 618 (47.5)	1 449 503 (47.2)	791 123 (48.1)	33 398 (47.9)
Race and ethnicity^a^				
American Indian or Alaska Native	26 038 (0.6)	15 612 (0.5)	10 296 (0.6)	1967 (2.8)
Asian	163 607 (3.4)	114 392 (3.7)	48 240 (2.9)	1967 (2.8)
Black	327 188 (6.9)	209 873 (6.8)	115 044 (7)	4757 (6.8)
Native Hawaiian or Pacific Islander	13 315 (0.3)	9011 (0.3)	4171 (0.3)	324 (0.5)
White	4 113 937 (86.7)	2 650 801 (86.3)	1 435 529 (87.3)	61 384 (88.1)
Other^b^	339 502 (7.2)	232 573 (7.6)	105 253 (6.4)	4191 (6.0)
Immunocompromised	1 529 622 (32.2)	976 334 (31.8)	542 896 (33.0)	24 188 (34.7)
Chronic lung disease	983 575 (20.7)	625 659 (20.4)	351 424 (21.4)	15 564 (22.3)
Cardiovascular disease	3 284 111 (69.2)	2 105 948 (68.6)	1 154 476 (70.2)	51 648 (74.1)

^a^ The choice of race and ethnicity allowed for more than 1 selection.

^b^ Includes individuals whose race and ethnicity designation was not included in the 1997 Office of Management and Budget minimum categories.

#### VE for Older Adults

**Figure 1.**
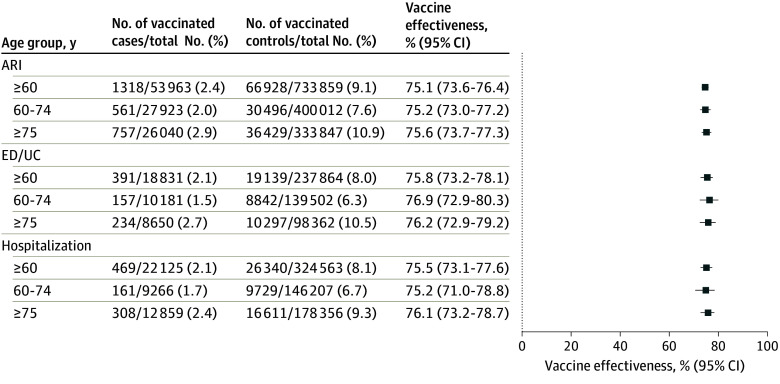
Estimated Vaccine Effectiveness Against Respiratory Syncytial Virus–Associated Medically Attended Respiratory Illness, Emergency Department or Urgent Care Visits, or Hospitalizations, October 1, 2023, to April 30, 2024 ARI indicates acute respiratory infection; ED/UC, emergency department or urgent care visit.

Of the total of 787 822 persons with ARI (53 963 cases and 733 859 controls), 1318 cases (2.4%) and 68 928 controls (9.1%) were vaccinated. The VE against RSV-associated medically attended respiratory illness for adults aged 60 years or older was 75.1% (95% CI, 73.6%-76.4%). VE was similar across age groups, and the VE was similar for emergency department or urgent care visits associated with RSV testing and for hospitalization for the overall group (Figure 1). VE by month and VE by state are shown in eTables 2 and 3 in Supplement 1.

#### VE for Immunocompromised Patients

**Figure 2.**
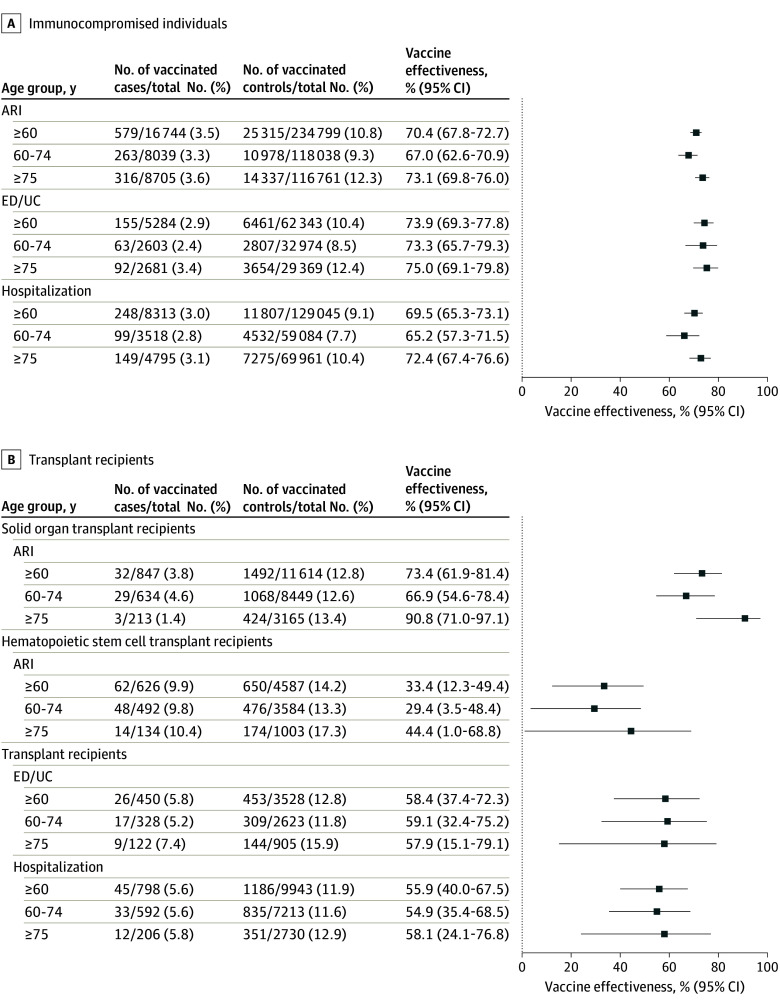
Estimated Vaccine Effectiveness Among Immunocompromised Individuals and Subgroups Against Respiratory Syncytial Virus–Associated Medically Attended Respiratory Illness, Emergency Department or Urgent Care Visits, or Hospitalizations, October 1, 2023, to April 30, 2024 A, Immunocompromised individuals. B, Transplant recipients include individuals with solid organ transplant or hematopoietic stem cell transplant. ARI indicates acute respiratory infection; ED/UC, emergency department or urgent care visit.

Figure 2A shows VE for immunocompromised patients. Among immunocompromised adults aged 60 years or older, the VE against RSV-associated medically attended respiratory illness was 70.4% (95% CI, 67.8%-72.7%), slightly lower for those aged 60 to 74 years (67.0% [95% CI, 62.6%-70.9%]) and higher for those aged 75 years or older (73.1% [95% CI, 69.8%-76.0%]). VE for hospitalized patients was substantial at 65.2% (95% CI, 57.3%-71.5%) for those aged 60 to 74 years and 72.4% (95% CI, 67.4%-76.6%) for those aged older than 75 years.

#### Transplant Recipients

Figure 2B shows the VE for transplant recipients, including both solid organ transplant recipients and hematopoietic stem cell transplant recipients. Generally, transplant recipients had lower VE compared with the group overall; VE against ARI was substantially lower for those who had a hematopoietic stem cell transplant (Figure 2B). Solid organ transplant recipients aged 60 y or older had VE against RSV-associated medically attended respiratory illness that was comparable to the group as a whole, but with a wider 95% CI, and VE was better for solid organ transplant recipients 75 years or older (Figure 2B).

Overall, among the 17 674 transplant recipients, VE against ARI ranged from 50% to 69% and from 54.9% to 58.1% for ARI-associated hospitalization. The lowest VE estimate was for the subset of 5213 patients who received a stem cell transplant (29.4%-44.4% VE for ARI).

#### Risk of GBS and ITP

Table 3 shows the risks of GBS and ITP after vaccination. For all vaccine recipients (64.7% [3 070 888 of 4 746 518] received the RSVPreF3+AS01 vaccine), there was an estimated excess of 11.2 GBS cases per 1 000 000 vaccines administered. The estimated excess of GBS cases associated with the RSVPreF (Abrysvo, Pfizer) vaccine was 18.2 per 1 000 000 vaccines administered, and for the RSVPreF3+AS01 vaccine (Arexvy, GBS), the estimated excess of GBS cases was 5.2 per 1 000 000 vaccinations. There was no excess risk of ITP after vaccination for RSV from either manufacturer.

**Table 3.** Risk of Guillain-Barré Syndrome and ITP After Respiratory Syncytial Virus Vaccination for Older Adults From July 1, 2023, to June 31, 2024

Risk	Cases during risk period, No.	Vaccines Administered, No.	IRR (95% CI)	Excess cases per 1 000 000 doses (95% CI)
Guillain-Barré syndrome				
Overall	102	4 746 518	2.1 (1.5 to 2.9)	11.2 (7.2 to 14.1)
RSVPreF3	51	3 070 888	1.5 (0.9 to 2.2)	5.2 (–0.9 to 9.2)
RSVPreF	51	1 643 827	2.4 (1.5 to 4.0)	18.2 (9.8 to 23.3)
ITP				
Overall	257	4 740 401	1.0 (0.9 to 1.2)	1.9 (–7.7 to 10.1)
RSVPreF3	171	3 067 030	1.1 (8.7 to 1.3)	3.7 (–8.4 to 13.5)
RSVPreF	84	1 641 602	0.9 (0.7 to 1.2)	–4.1 (–22.5 to 9.7)

Abbreviations: IRR, incidence rate ratio; ITP, immune thrombocytopenic purpura.

## Discussion

More than 10.6 million adults aged 60 years or older received a newly licensed RSV protein subunit vaccine approved before the 2023-2024 respiratory virus season.^9^ For this season, the Centers for Disease Control and Prevention public health surveillance documented a typical RSV pattern similar to pre–COVID-19 pandemic seasons with a peak in hospitalizations during the last week of December. Our EHR-associated RSV laboratory testing trends precisely mirrored public health surveillance trends of RSV circulation, demonstrating its fitness for the purpose of studying RSV-related events among older adults. Our overall estimates for VE are based on 787 822 patients aged 60 years or older who were tested for RSV, and our safety analyses are based on 4 746 518 vaccine recipients drawn from the large Cosmos population that reflects the demographics reported in the US Census.^5^ The large sample size allowed us to evaluate important subpopulations of patients for VE and characterize the frequency of rare adverse events. Our data confirm that, during the first year after licensure, vaccines against RSV based on protein subunits of the virus are effective in persons aged 60 years or older. A VE of approximately 75% was similar for both patients aged 60 to 74 years and those aged 75 years or older against all outcomes: ARI-associated encounters overall, ARI-associated emergency or urgent care visits, or ARI-associated hospitalization. These estimates are comparable to the prior analyses performed with smaller populations (the largest prior report contained 293 704 total participants) or more restricted populations (eg, a study of Medicare patients with end-stage kidney disease).^10^ During prelicensure clinical trials, the VE was reported to be 67% to 86%,^1,2^ and during early postlicensure studies, VE was reported to be 73% to 82%, but with wide 95% CIs that ranged from 41% to 95%.^10^ Because our population was deidentified, we could not investigate the overlap of our population with those included in prior studies. The use of Cosmos permits analysis of aggregated deidentified data from health care organizations that use the widely used Epic Corporation EHR with minimal lag time (several weeks); in contrast, labor-intensive clinical trial networks or observational studies require inspection of individual patient records that may delay access to results for months or longer.

Our sample included 251 543 immunocompromised patients tested for RSV. Among immunocompromised patients overall, VE ranged from approximately 67% to 73% for ARI and from approximately 65% to 72% for hospitalization, for both age groups, indicating that substantial protection is conferred by the vaccine. However, among the subset of 17 674 transplant recipients, VE against ARI ranged from 50% to 69% and from 55% to 58% for ARI-associated hospitalization. The lowest VE estimate was for the subset of 5213 patients who received a stem cell transplant (29%-44% VE for ARI). Immunocompromised patients overall, except for those with stem cell transplants, still achieved substantial protection from RSV vaccination.

VE for ARI overall approximated VE for ARI-associated hospitalizations in our study and in those previously published.^10,11^ Hospitalization might represent more severe disease but may also be a setting more likely to prompt RSV testing in this age group, and the indication for hospitalization cannot be ascertained. The adult population in our case-control study had high prevalence of comorbid conditions, including 69.9% with cardiovascular disease and 20.9% with chronic lung disease, which were comparable for each vaccine manufacturer. The additional stress of RSV infection might adversely affect underlying heart disease or lung disease,^12^ and the need for hospitalization may reflect the complex of diseases in older individuals.

Early safety data for these vaccines during both clinical trials and initial postlicensure analyses have suggested a possible association between ITP and GBS, but the more modest sample sizes available for these analyses made these suspicions inconclusive. In the 90 days after vaccination, 102 cases of GBS and 225 cases of ITP were recorded in the EHR of more than 4.6 million vaccinated adults. Excess cases that occurred during the 6 weeks after vaccination (days 1-42) were compared with the baseline rate that occurred during the subsequent 6 weeks (days 43-90). Vaccinated adults did not have more ITP diagnoses in the 6 weeks after vaccination compared with the subsequent period. However, for GBS in a population in which approximately two-thirds received the RSVPreF3+AS01 vaccine (as observed among the study’s vaccine recipients), an estimated 11.2 cases per 1 000 000 patients in excess of those expected based on the control period was observed in the first 6 weeks compared with those in days 43 to 90.

Further stratification by vaccine manufacturer resulted in an estimated 5.2 excess GBS cases per 1 000 000 vaccine recipients for the RSVPreF3+AS01 vaccine. However, the 95% CI for this estimate included no elevated risk. There was an excess of 18.2 GBS cases per 1 000 000 recipients of the RSVPreF vaccine.

### Limitations

Several limitations exist for this study. Cosmos has information about vaccine administration from both administration events at the health care organization, through patient reports of vaccination outside of the health care organization, and from external sources, such as health care organizations not contributing to Cosmos and state immunization registries through electronic health information exchanges. Despite this effort, it is still possible that some vaccinated patients did not have RSV vaccination recorded in the EHR. Given that the proportion of vaccinated patients in our study mirrors the coverage in the VE study populations (5%-10%),^10^ the effect of such an error is likely small. Also, misclassification of vaccinated patients as unvaccinated would likely reduce our estimates of VE, which are substantial and consistent with prior reports. In addition, care-seeking behaviors between vaccine recipients and nonrecipients may differ, but the use of a test-negative case-control design aims to minimize this potential confounding factor. The test-negative case-control design is intended to minimize the limitation that we study only those who seek care and are tested for a respiratory infection, but it is still possible that unattended infections would affect the results. We accepted both nucleic acid and antigen detection tests to diagnose RSV. Diagnostic testing for RSV using real-time reverse transcription–polymerase chain reaction tests is highly sensitive. Antigen detection tests for adults are less sensitive, which could result in misclassification of cases as controls and an underestimation of VE. Another concern might be the confounding effect of other diseases in the older population. Although we did not match for comorbidities, the case and control populations did not differ substantially in baseline comorbidities, and VE resembled values derived from smaller studies. Also, any EHR study is subject to the possibilities of underdiagnosis, overdiagnosis, or misdiagnosis. However, establishing the cohort with a laboratory test for RSV during a respiratory infection minimizes this risk.

Another potential limitation is the precision of the diagnosis of GBS. Although it is not possible to ascertain that the GBS diagnoses meet the criteria described in Sejvar et al^13^ because of the aggregated deidentified data (which precludes confirmatory chart review), the discrepancy in vaccine-associated GBS estimates between vaccine manufacturers should not be affected by the inability to apply the strict application of the GBS case definition. Moreover, we cannot ascertain the cause of hospitalization, only that it occurred during an admission in which RSV was at least suspected. In this patient population with many serious underlying diseases, it is likely that hospitalization may be attributable to the underlying comorbidities. The implications of a diagnosis associated with immunocompromise may vary, for some patients with a diagnosis ordinarily treated with immunosuppressive drugs may not be so treated, or the degree of immunocompromise may be small. These possible errors would tend to overestimate the VE for immunocompromised patients. We also must consider that although the data platform from which our data were derived is very large, it contains the records of those with contact with a health organization that uses the Epic EHR and may not be representative of all US residents. Lastly, our retrospective observational study can identify associations but not causation.

## Conclusions

By leveraging the power of real-time access to data in clinical practice on a data platform with more than 270 million patient records, this study provides previously unavailable estimates of VE for patient subgroups and additional safety data concerning rare adverse events. The VE was slightly diminished among immunocompromised patients overall, more so among the subset of patients who had received transplants, and the protection after stem cell transplant was limited. The risk of immune thrombocytopenic purpura after vaccination was not elevated, but the risk of GBS was statistically significantly elevated in patients who received the RSVPreF vaccine but not in those who received RSVPreF3+AS01 vaccine, although the risk was small. These observations should inform clinicians’ choices and patient instructions.

## Notes

Supplement 1.
eMethods.

eReferences.
**eFigure 1.** RSV-Associated Hospitalizations Among Older Adults, 2023-24 RSV Season **eFigure 2.** RSV Percent Positive Rate, 2023-24 RSV Season **eTable 1.** Baseline Characteristics for Vaccine Effectiveness Analysis by Month **eTable 2.** Vaccine Effectiveness Against RSV-Associated Medically Attended Respiratory Illness by Month, Adults ≥60 **eTable 3.** Vaccine Effectiveness Against RSV-Associated Medically Attended Respiratory Illness by State, Adults ≥60 **eTable 4.** Diagnosis and Procedure Codes Used in Analyses

Supplement 2.
Data Sharing Statement
