# Supplementary material for: Effectiveness and Safety of Respiratory Syncytial Virus Vaccine for US Adults Aged 60 Years or Older
Source: JAMA Netw Open. 2025 May 9;8(5):e258322. doi: 10.1001/jamanetworkopen.2025.8322 (PMC12065041; doi:10.1001/jamanetworkopen.2025.8322)
Supplement: Supplement 1. — eMethods. eReferences. eFigure 1. RSV-Associated Hospitalizations Among Older Adults, 2023-24 RSV Season eFigure 2. RSV Percent Positive Rate, 2023-24 RSV Season eTable 1. Baseline Characteristics for Vaccine Effectiveness Analysis by Month eTable 2. Vaccine Effectiveness Against RSV-Associated Medically Attended Respiratory Illness by Month, Adults ≥60 eTable 3. Vaccine Effectiveness Against RSV-Associated Medically Attended Respiratory Illness by State, Adults ≥60 eTable 4. Diagnosis and Procedure Codes Used in Analyses [file jamanetwopen-e258322-s001.pdf]

Supplemental Online Content

Effectiveness and Safety of Respiratory Syncytial Virus Vaccine for US Adults Aged 60 Years or Older

Fry SE, Terebuh P, Kaelber DC, et al. Effectiveness and safety of respiratory syncytial virus vaccine for US adults aged 60 years or older. *JAMA Netw Open*. 2025;8(5):e258322. doi:10.1001/jamanetworkopen.2025.8322

eMethods.

eFigure 1. RSV-Associated Hospitalizations Among Older Adults, 2023-24 RSV Season

eFigure 2. RSV Percent Positive Rate, 2023-24 RSV Season

eTable 1. Baseline Characteristics for Vaccine Effectiveness Analysis by Month

eTable 2. Vaccine Effectiveness Against RSV-Associated Medically Attended Respiratory Illness by Month, Adults ≥60

eTable 3. Vaccine Effectiveness Against RSV-Associated Medically Attended Respiratory Illness by State, Adults ≥60

eTable 4. Diagnosis and Procedure Codes Used in Analyses

This supplemental material has been provided by the authors to give readers additional information about their work.

© 2025 Fry SE et al. *JAMA Network Open.*

eMethods

1. Evaluation of RSV-associated hospitalizations among adults ≥60 years old during the 2023-24 RSV season

First, we used Cosmos data to evaluate the number of RSV-associated hospitalizations among adults ≥60 years old during the 2023-24 RSV season (eFigure 1). We included inpatient encounters for adults ≥60 years that were associated with a positive RSV test (10 days before to 3 days after first day of hospitalization) and an acute respiratory infection diagnosis (ascertained using the same ICD-10 codes as the main analysis). We compared these results to those from CDC public health surveillance. The CDC's RSV-NET conducts population-based surveillance for hospitalizations associated with laboratory-confirmed RSV, using data collected from acute-care hospital facilities in 12 states.[1] RSV-NET calculates the weekly rate of RSV-associated hospitalizations per 100,000 people. Our results show that Cosmos-ascertained weekly rates for RSV-associated hospitalizations closely mirror those reported by RSV-NET.

2. Evaluation of the percent positive rate for RSV laboratory tests during the 2023-24 RSV season

Next, we used Cosmos data to evaluate the percent positive rate for RSV tests during the 2023-24 RSV season (eFigure 2). This was calculated by dividing the number of positive RSV tests per week by the total number of tests. We compared these estimates to those from the CDC'sNational Respiratory and Enteric Virus Surveillance System (NREVSS).[2] Given that NREVSS obtains data from reports from laboratories, it is not able to report a percent positive rate among specific age groups. Using Cosmos, we evaluated the percent positive rate both for people of all ages (to allow comparison with NREVSS data) and among adults aged ≥60 years. NREVSS reported a peak RSV percent positive rate of 12.5% among all ages the week of 11/25/23. Among all ages in Cosmos, the peak RSV percent positive rate was 12.6% and occurred the week of 11/22/23-11/28/23. We were also able to evaluate the rate specifically among adults ≥60 years old, and found that the peak percent positive rate in this age group was 6.6% and occurred the week of 11/29/23-12/5/23.

References

1. Centers for Disease Control and Prevention . RSV-NET: respiratory syncytial virus hospitalization surveillance network. 2023. Available: https://www.cdc.gov/rsv/research/rsv-net/dashboard.html
2. Centers for Disease C. National Respiratory and Enteric Virus Surveillance System (NREVSS). https://www.cdc.gov/surveillance/nrevss/.

© 2025 Fry SE et al. *JAMA Network Open.*

eFigure 1. RSV-Associated Hospitalizations among Older Adults, 2023-2024 RSV Season

a. Estimates from CDC RSV-NET: Weekly Rates of RSV-Associated Hospitalizations per 100,000 among Adults ≥65 years old

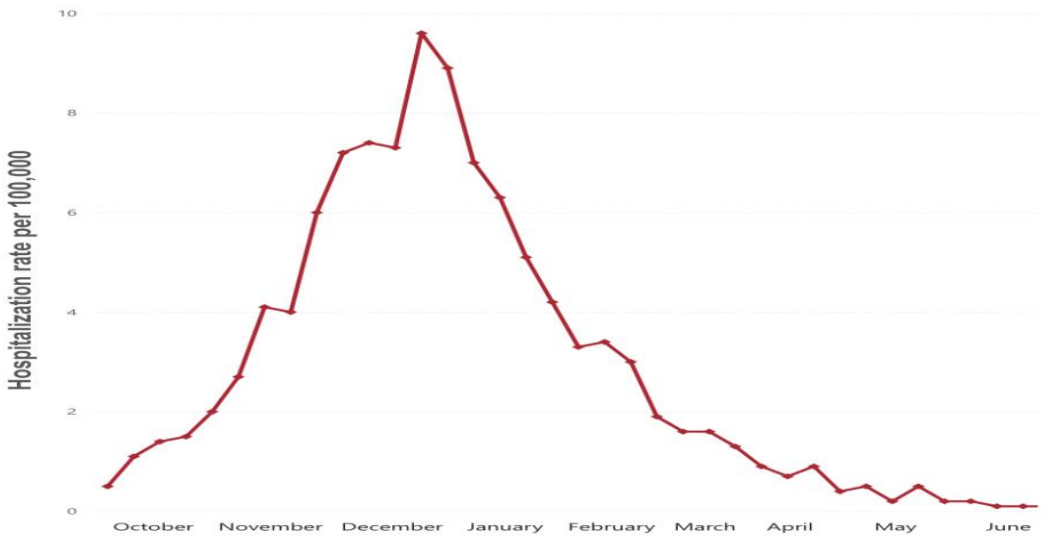

b. Estimates from Epic Cosmos - Weekly count of RSV-Associated Hospitalizations among Adults ≥60 years old

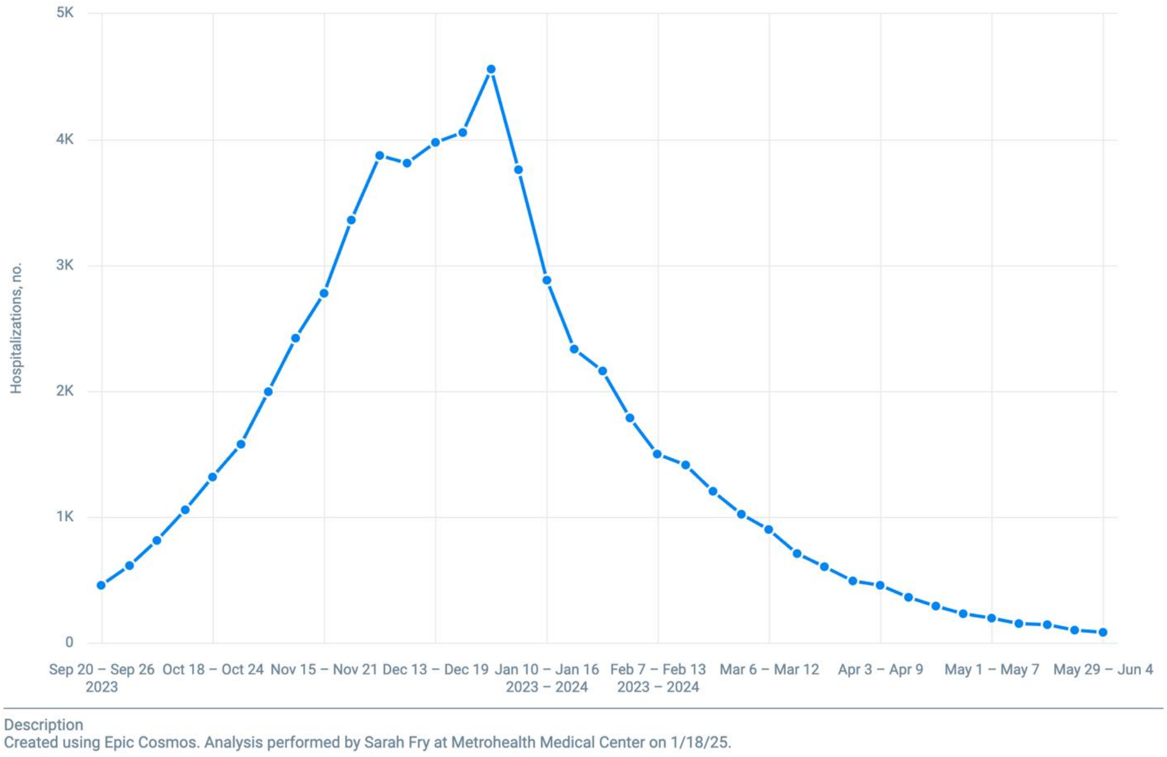

© 2025 Fry SE et al. *JAMA Network Open.*

eFigure 2. RSV Percent Positive Rate, 2023-2024 RSV Season

a. Percent Positive Rates from CDC's National Respiratory and Enteric Virus Surveillance System (NREVSS) among All Ages

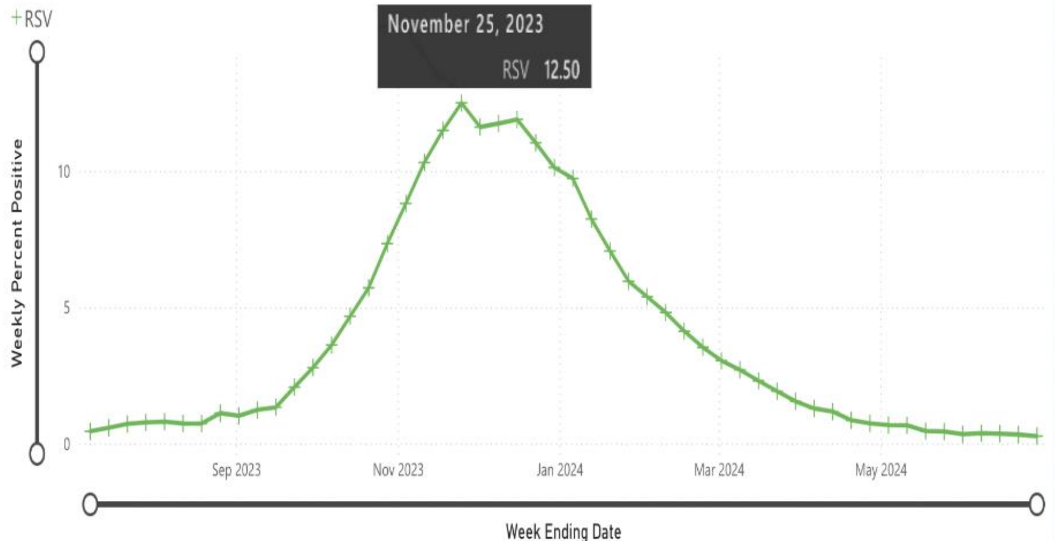

b. Percent Positive Rates Obtained from Epic Cosmos among Adults Aged ≥60 years

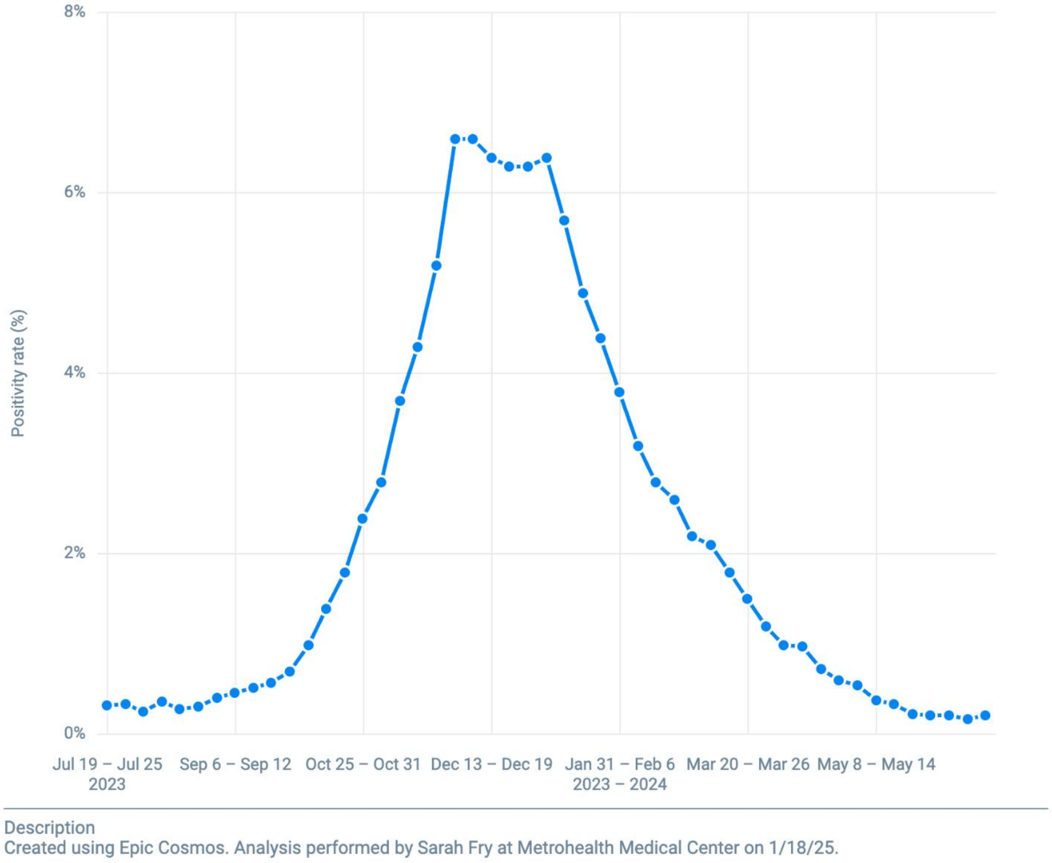

© 2025 Fry SE et al. *JAMA Network Open.*

c. Percent Positive Rates Obtained from Epic Cosmos among All Ages

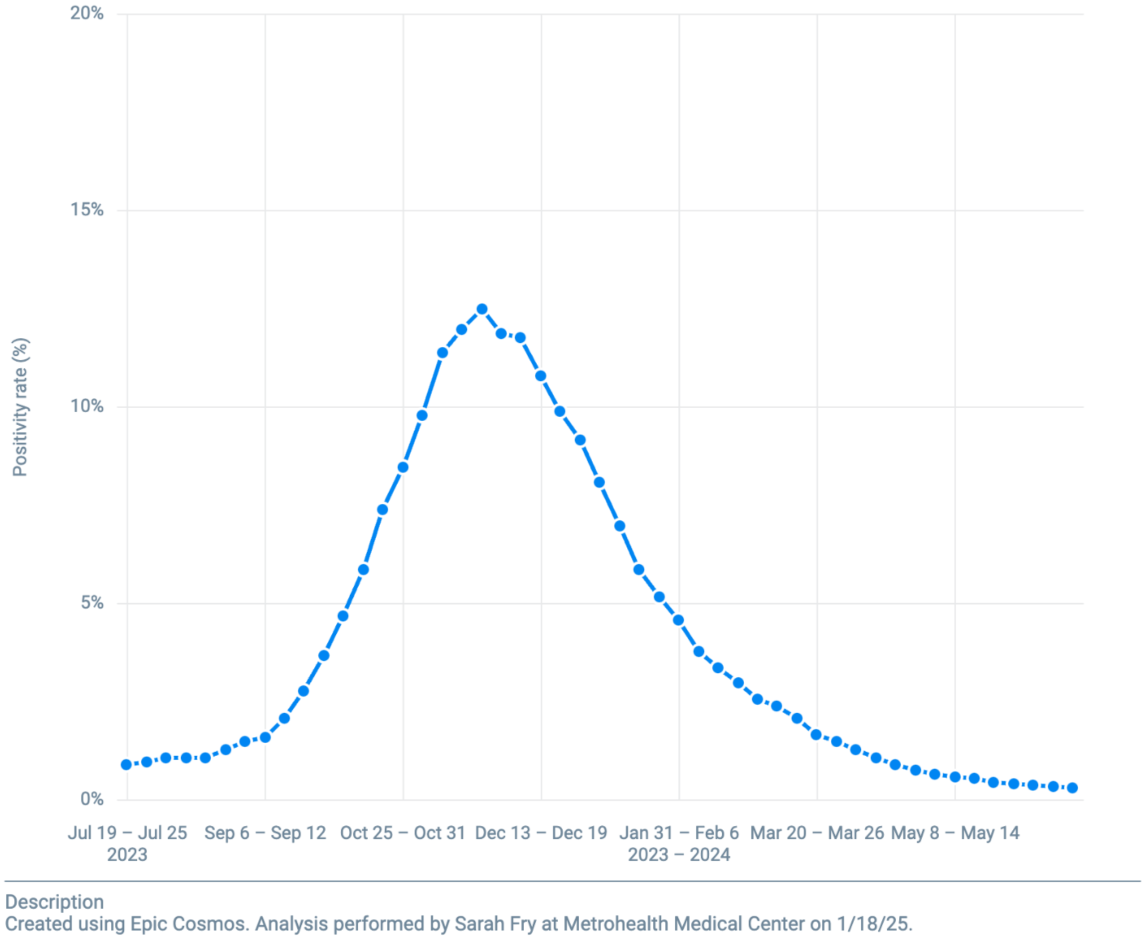

© 2025 Fry SE et al. *JAMA Network Open.*

eTable 1. Baseline Characteristics for Vaccine Effectiveness Analysis by Month

a. October

	Cases	Controls
	n=2,593	n=89,588
Median age, y – no. (IQR)	73 (67-81)	74 (67-81)
Race – no. (%)		
White	1,910 (73.7)	73,190 (81.7)
Black	486 (18.7)	11982 (13.4)
Asian	96 (3.7)	2023 (2.3)
American Indian and Alaska Native	21 (0.81)	782 (0.87)
Native Hawaiian and Pacific Islander	12 (0.46)	278 (0.31)
Other	282 (10.9)	7,033 (7.9)
Immunocompromised – no. (%)	735 (28.3)	29,884 (33.4)
Chronic lung disease – no. (%)	735 (28.3)	28,809 (32.2)
Cardiovascular disease – no. (%)	1,746 (67.3)	64839 (72.4)

b. November

	Cases	Controls
	n=8,613	n=112,593
Median age, y – no. (IQR)	74 (67-81)	74 (67-81)
Race – no. (%)		
White	6,785 (78.8)	92,048 (81.8)
Black	1,291 (15)	14,948 (13.3)
Asian	270 (3.1)	2,594 (2.3)
American Indian and Alaska Native	69 (0.80)	975 (0.87)
Native Hawaiian and Pacific Islander	31 (0.36)	353 (0.31)
Other	887 (10.3)	9,119 (8.1)
Immunocompromised – no. (%)	2,439 (28.3)	36,021 (32.0)
Chronic lung disease – no. (%)	2,530 (29.4)	34,653 (30.8)
Cardiovascular disease – no. (%)	6,015 (69.8)	80,478 (71.5)

c. December

© 2025 Fry SE et al. *JAMA Network Open.*

	Cases	Controls
	n=19,715	n=179,169
Median age, y – no. (IQR)	74 (67-82)	73 (67-81)
Race – no. (%)		
White	16472 (83.6)	145653 (81.3)
Black	2255 (11.4)	24,754 (13.8)
Asian	492 (2.5)	4,040 (2.3)
American Indian and Alaska Native	146 (0.74)	1472 (0.82)
Native Hawaiian and Pacific Islander	59 (0.30)	524 (0.29)
Other	1,627 (8.3)	14,077 (7.9)
Immunocompromised – no. (%)	5,838 (29.6)	53,808 (30.0)
Chronic lung disease – no. (%)	5,730 (29.1)	52,035 (29)
Cardiovascular disease – no. (%)	13,976 (70.9)	125,687 (70.1)

d. January

	Cases	Controls
	n=16,332	n=168,631
Median age, y – no. (IQR)	75 (68-83)	73 (67-81)
Race – no. (%)		
White	14,156 (86.7)	136,021 (80.7)
Black	1,496 (9.2)	13,768 (8.2)
Asian	333 (2.0)	3,933 (2.3)
American Indian and Alaska Native	138 (0.85)	1,482 (0.88)
Native Hawaiian and Pacific Islander	43 (0.26)	469 (0.28)
Other	1,049 (6.4)	13,768 (8.2)
Immunocompromised – no. (%)	4,929 (30.2)	52,210 (31.0)
Chronic lung disease – no. (%)	4,814 (29.5)	50,855 (30.2)
Cardiovascular disease – no. (%)	11,869 (72.7)	119,887 (71.1)

e. February

	Cases	Controls
	n=7,183	n=134,112
Median age, y – no. (IQR)	74 (67-82)	73 (67-81)

© 2025 Fry SE et al. *JAMA Network Open.*

Race – no. (%)		
White	6,202 (86.3)	108,993 (81.3)
Black	632 (8.8)	10,263 (7.7)
Asian	153 (2.1)	2,967 (2.2)
American Indian and Alaska Native	64 (0.89)	1,172 (0.87)
Native Hawaiian and Pacific Islander	17 (0.24)	358 (0.27)
Other	457 (6.4)	10263 (7.7)
Immunocompromised – no. (%)	2,240 (31.2)	42,553 (31.7)
Chronic lung disease – no. (%)	565 (30.9)	24,981 (31.1)
Cardiovascular disease – no. (%)	1,283 (70.3)	56,972 (70.8)

f. March

	Cases	Controls
	n=3,884	n=122,072
Median age, y – no. (IQR)	75 (68-83)	73 (67-81)
Race – no. (%)		
White	3,394 (87.4)	98,944 (81.1)
Black	302 (7.8)	16,828 (13.8)
Asian	84 (2.2)	2,927 (2.4)
American Indian and Alaska Native	41 (1.1)	1,125 (0.92)
Native Hawaiian and Pacific Islander	13 (0.34)	385 (0.32)
Other	241 (6.2)	9,766 (8.0)
Immunocompromised – no. (%)	1,216 (31.3)	40,032 (32.8)
Chronic lung disease – no. (%)	1,204 (31.0)	39,533 (32.4)
Cardiovascular disease – no. (%)	2,781 (71.6)	88,214 (72.3)

g. April

	Cases	Controls
	n=1,627	n=105,033
Median age, y – no. (IQR)	75 (68-82)	74 (67-81)
Race – no. (%)		
White	1,406 (86.4)	85,251 (81.2)

© 2025 Fry SE et al. *JAMA Network Open.*

Black	146 (9.0)	14,054 (13.4)
Asian	26 (1.6)	2,693 (2.6)
American Indian and Alaska Native	12 (0.74)	994 (0.95)
Native Hawaiian and Pacific Islander	N/A	357 (0.34)
Other	107 (6.6)	8,742 (8.3)
Immunocompromised – no. (%)	503 (30.9)	35,500 (33.8)
Chronic lung disease – no. (%)	501 (31.0)	34,833 (33.2)
Cardiovascular disease – no. (%)	1,196 (73.5)	76,766 (73.1)

eTable 2. Vaccine Effectiveness against RSV-Associated Medically Attended Respiratory Illness by Month, Adults ≥60

State	Group	Exposed	Unexposed	Odds Ratio	Vaccine Effectiveness	Test Positivity (%)
October	Cases	18	2,575	0.254	74.63%	1.6%
	Controls	2,402	87,186			
November	Cases	109	8,504	0.213	78.70%	4.4%
	Controls	6,392	106,201			
December	Cases	382	19,333	0.220	77.97%	6.6%
	Controls	14,749	164,420			
January	Cases	493	15,839	0.281	71.91%	5.7%
	Controls	16,824	151,807			
February	Cases	272	6,911	0.291	70.94%	3.1%
	Controls	15,996	118,116			
March	Cases	186	3,698	0.329	67.12%	1.8%
	Controls	16,196	105,876			
April	Cases	102	1,525	0.404	59.57%	0.9%
	Controls	14,910	90,123			

eTable 3. Vaccine Effectiveness against RSV-Associated Medically Attended Respiratory Illness by State, Adults ≥60

State	Group	Exposed	Unexposed	Odds Ratio	Vaccine Effectiveness	Test Positivity (%)
Arizona	Cases	17	540	0.256	74.39%	3.9%
	Controls	810	6,589			
Arkansas	Cases	16	359	0.465	53.52%	4.0%
	Controls	604	6,299			

© 2025 Fry SE et al. *JAMA Network Open.*

California	Cases	55	1,582	0.255	74.48%	4.0%
	Controls	2,776	20,375			
Colorado	Cases	73	1,623	0.198	80.18%	4.7%
	Controls	4,469	19,694			
Connecticut	Cases	39	1,811	0.189	81.08%	4.9%
	Controls	2,235	19,635			
Florida	Cases	75	3,835	0.225	77.53%	3.5%
	Controls	5,505	63,239			
Georgia	Cases	34	1,430	0.237	76.34%	3.9%
	Controls	2,262	22,514			
Illinois	Cases	82	3,357	0.226	77.36%	4.5%
	Controls	4,298	39,845			
Indiana	Cases	82	3,357	0.249	75.12%	6.1%
	Controls	1,615	16,448			
Iowa	Cases	35	638	0.267	73.35%	3.9%
	Controls	1,802	8,755			
Kansas	Cases	15	341	0.239	76.06%	4.2%
	Controls	807	4,392			
Kentucky	Cases	35	1,232	0.270	72.98%	5.1%
	Controls	1,476	14,039			
Maryland	Cases	14	1,019	0.147	85.29%	4.1%
	Controls	1,244	13,321			
Massachusetts	Cases	32	1,142	0.217	78.29%	4.9%
	Controls	1,626	12,600			
Michigan	Cases	91	1,753	0.340	65.96%	4.2%
	Controls	3,338	21,887			
Minnesota	Cases	67	1,564	0.270	72.97%	4.3%
	Controls	3,026	19,091			
Missouri	Cases	14	505	0.289	71.09%	4.4%
	Controls	586	6,110			
Nebraska	Cases	13	421	0.187	81.33%	4.7%
	Controls	792	4,789			
Nevada	Cases	15	350	0.316	68.42%	4.8%
	Controls	637	4,694			
New Jersey	Cases	13	567	0.301	69.89%	5.2%
	Controls	497	6,527			
New York	Cases	122	4,845	0.223	77.71%	4.9%
	Controls	6,190	54,799			
North Carolina	Cases	77	2,645	0.258	74.23%	4.1%
	Controls	6,190	54,799			
Ohio	Cases	110	4,364	0.198	80.20%	4.6%
	Controls	6,850	53,795			
Oregon	Cases	11	311	0.247	75.30%	3.7%
	Controls	566	3,953			
Pennsylvania	Cases	103	5,140	0.200	79.95%	4.8%

© 2025 Fry SE et al. *JAMA Network Open.*

	Controls	5,812	58,145			
South Carolina	Cases	35	2,002	0.268	73.21%	4.4%
	Controls	1,820	27,885			
South Dakota	Cases	22	212	0.485	51.46%	4.3%
	Controls	602	2,816			
Tennessee	Cases	15	668	0.318	68.22%	5.0%
	Controls	547	7,741			
Texas	Cases	51	2,859	0.245	75.52%	3.9%
	Controls	2,996	41,109			
Utah	Cases	11	143	0.340	66.04%	3.8%
	Controls	499	2,203			
Virginia	Cases	39	2,145	0.207	79.35%	4.8%
	Controls	2,272	25,806			
Washington	Cases	18	248	0.341	65.93%	3.6%
	Controls	687	3,225			
West Virginia	Cases	22	1,062	0.216	78.41%	4.7%
	Controls	1,237	12,893			
Wisconsin	Cases	94	2,460	0.238	76.25%	5.6%
	Controls	3,896	24,217			

eTable 4. Diagnosis and Procedure Codes Used in Analyses

a. **Acute respiratory illness**

ICD-10-CM	Description
J00	Acute nasopharyngitis (common cold)
J01	Acute sinusitis
J02	Acute pharyngitis
J03	Acute tonsillitis
J04	Acute laryngitis and tracheitis
J05	Acute obstructive laryngitis (croup) and epiglottitis
J06	Acute upper respiratory infections of multiple and unspecified sites
J09	Influenza due to certain identified influenza viruses
J10	Influenza due to other identified influenza virus
J11	Influenza, virus not identified
J12	Viral pneumonia, not elsewhere classified
J13	Pneumonia due to Streptococcus pneumoniae
J14	Pneumonia due to Haemophilus influenzae
J15	Bacterial pneumonia, not elsewhere classified
J16	Pneumonia due to other infectious organisms, not elsewhere classified

© 2025 Fry SE et al. *JAMA Network Open.*

J17	Pneumonia in diseases classified elsewhere
J18	Pneumonia, organism unspecified
J20	Acute bronchitis
J21	Acute bronchiolitis
J22	Unspecified acute lower respiratory infection
R05	Cough

b. Immunocompromised status

ICD-10-CM Code	Description
B20	HIV disease resulting in infectious and parasitic diseases
C00-C96	Malignant neoplasms
D46	Myelodysplastic syndromes
D61	Other aplastic anemias and other bone marrow failure syndromes
D70	Neutropenia
D71	Functional disorders of polymorphonuclear neutrophils
D80	Immunodeficiency with predominantly antibody defects
D81	Combined immunodeficiencies
D82	Immunodeficiency associated with other major defects
D83	Common variable immunodeficiency
D84	Other immunodeficiencies
D86	Sarcoidosis
D89	Other disorders involving the immune mechanism, not elsewhere classified
E85	Amyloidosis
G35	Multiple sclerosis
L93	Lupus erythematosus
L40.5	Arthropathic psoriasis
L94	Other localized connective tissue disorders
M05	Rheumatoid arthritis with rheumatoid factor
M06	Other rheumatoid arthritis
M07	Enteropathic arthropathies
M30	Polyarteritis nodosa and related conditions

© 2025 Fry SE et al. *JAMA Network Open.*

ICD-10-CM Code	Description
M31	Other necrotizing vasculopathies
M32	Systemic lupus erythematosus (SLE)
M33	Dermatopolymyositis
M34	Systemic sclerosis (scleroderma)
M35	Other systemic involvement of connective tissue
M46	Other inflammatory spondylopathies
K70	Alcoholic liver disease
K72	Hepatic failure, not elsewhere classified
K74	Fibrosis and cirrhosis of liver
N04	Nephrotic syndrome
R18	Ascites
Z48.2	Encounter for aftercare following organ transplant
Z94	Transplanted organ and tissue status
D47.Z1	Post-transplant lymphoproliferative disorder (PTLD)
T86	Complications of transplanted organs and tissue

c. Chronic lung disease

ICD-10-CM	Description
J41	Simple and mucopurulent chronic bronchitis
J42	Unspecified chronic bronchitis
J43	Emphysema
J44	Other chronic obstructive pulmonary disease (COPD)
J45	Asthma
J47	Bronchiectasis

d. Cardiovascular disease

ICD-10-CM	Description
I10	Essential (primary) hypertension
I20	Angina pectoris
I21	Acute myocardial infarction
I22	Subsequent myocardial infarction

© 2025 Fry SE et al. *JAMA Network Open.*

I23	Certain current complications following acute MI
I24	Other acute ischemic heart diseases
I25	Chronic ischemic heart disease
I26	Pulmonary embolism
I30	Acute pericarditis
I31	Other diseases of pericardium
I32	Pericarditis in diseases classified elsewhere
I33	Acute and subacute endocarditis
I34	Nonrheumatic mitral valve disorders
I35	Nonrheumatic aortic valve disorders
I36	Nonrheumatic tricuspid valve disorders
I37	Pulmonary valve disorders
I38	Endocarditis, valve unspecified
I39	Endocarditis and heart valve disorders in diseases
I40	Acute myocarditis
I41	Myocarditis in diseases classified elsewhere
I42	Cardiomyopathy
I43	Cardiomyopathy in diseases classified elsewhere
I44	Atrioventricular and left bundle-branch block
I45	Other conduction disorders
I46	Cardiac arrest
I47	Paroxysmal tachycardia
I48	Atrial fibrillation and flutter
I49	Other cardiac arrhythmias
I51	Complications and ill-defined heart disease
I70	Atherosclerosis
I71	Aortic aneurysm and dissection
I72	Other aneurysm
I73	Other peripheral vascular diseases
I74	Arterial embolism and thrombosis
I75	Atheroembolism
I76	Septic arterial embolism

© 2025 Fry SE et al. *JAMA Network Open.*

I77	Other disorders of arteries and arterioles
I78	Diseases of capillaries
I79	Disorders of arteries, arterioles, and capillaries in diseases classified elsewhere

e. Solid organ transplant

ICD-10-CM	
T86.1	Complications of kidney transplant
T86.2	Complications of heart transplant
T86.3	Complications of heart-lung transplant
T86.4	Complications of liver transplant
T86.81	Complications of lung transplant
T86.83	Complications of bone graft
T86.84	Complications of corneal transplant
T86.85	Complications of intestine transplant
T86.89	Complications of other transplanted tissue
T86.9	Complications of unspecified transplanted organ and tissue
Z48.2	Encounter for aftercare following organ transplant
Z94	Transplanted organ and tissue status
D47.Z1	Post-transplant lymphoproliferative disorder (PTLD)
CPT-4	
32851	Lung transplant (1 lung) without cardiopulmonary bypass
32852	Lung transplant (1 lung) with cardiopulmonary bypass
32853	Lung transplant (2 lungs) without cardiopulmonary bypass
32854	Lung transplant (2 lungs) with cardiopulmonary bypass
33935	Heart-lung transplant with recipient cardiectomy and pneumonectomy
33945	Heart transplant with or without recipient cardiectomy
44135	Intestinal allotransplantation from cadaver donor
47135	Orthotopic liver allotransplantation, partial or whole, any age
48160	Pancreatectomy with pancreas/islet cell transplantation
48554	Transplantation of pancreatic allograft
50360	Renal allotransplantation without recipient nephrectomy
Healthcare Common Procedure Coding System	

© 2025 Fry SE et al. *JAMA Network Open.*

S2060	Lobar lung transplantation
S2065	Simultaneous pancreas-kidney transplant
S2152	Solid organ transplant package
02YA0Z0	Transplantation of heart, allogeneic, open approach
02YA0Z1	Transplantation of heart, syngeneic, open approach
02YA0Z2	Transplantation of heart, zooplastic, open approach
0FY00Z0	Transplantation of liver, allogeneic, open approach
0FY00Z1	Transplantation of liver, syngeneic, open approach
0FY00Z2	Transplantation of liver, zooplastic, open approach
0DBO0ZZ	Transplantation of pancreas, allogeneic, open approach
0DBQ0ZZ	Transplantation of pancreas, syngeneic, open approach
0DBP0ZZ	Transplantation of stomach, allogeneic, open approach
0BPS0ZZ	Transplantation of left lung, allogeneic, open approach
0BPT0Z1	Transplantation of left lung, syngeneic, open approach
0BPU0ZZ	Transplantation of right lung, syngeneic, open approach
0BPR0ZZ	Transplantation of right lung, zooplastic, open approach
0BPT0ZZ	Transplantation of right lung, allogeneic, open approach
0BQQ0ZZ	Transplantation of bilateral lungs, allogeneic, open approach
0BPB0ZZ	Transplantation of left lower lung lobe, allogeneic, open approach
0BPQ0ZZ	Transplantation of right lower lung lobe, allogeneic, open approach
0BPR0ZZ	Transplantation of right middle lung lobe, allogeneic, open approach
0BPS0ZZ	Transplantation of right upper lung lobe, allogeneic, open approach
0D4G0ZZ	Transplantation of small intestine, allogeneic, open approach
0DBJ0ZZ	Transplantation of large intestine, allogeneic, open approach
0T1P0ZZ	Transplantation of left kidney, syngeneic, open approach
0T1R0ZZ	Transplantation of left kidney, allogeneic, open approach
0T1S0ZZ	Transplantation of left kidney, zooplastic, open approach
0T1V0ZZ	Transplantation of right kidney, syngeneic, open approach
0T1T0ZZ	Transplantation of right kidney, allogeneic, open approach
0T1W0ZZ	Transplantation of right kidney, zooplastic, open approach
0T1U0ZZ	Transplantation of other body system into POC, via opening

© 2025 Fry SE et al. *JAMA Network Open.*

0QDP0ZZ	Transplantation of ear, nose & sinus into POC, percutaneous approach
0QDR0ZZ	Transplantation of other body system into POC, percutaneous endoscopic approach

f. Hematopoietic stem cell transplant

ICD-10-CM	
T86.0	Complications of bone marrow transplant
T86.02	Bone marrow transplant failure
T86.03	Bone marrow transplant infection
T86.01	Bone marrow transplant rejection
T86.09	Other complications of bone marrow transplant
T86.00	Unspecified complication of bone marrow transplant
Z94.81	Bone marrow transplant status
Z94.84	Stem cells transplant status
T86.5	Complications of stem cell transplant
CPT-4	
38240	Hematopoietic Progenitor Cell (HPC) Transplantation; Allogeneic Transplant
HCPCS	
S2150	Bone marrow or blood-derived stem cells (peripheral or umbilical), allogeneic or autologous, harvesting, transplantation, and related complications

© 2025 Fry SE et al. *JAMA Network Open.*
