# Supplementary material for: Effectiveness and Safety of Respiratory Syncytial Virus Vaccine for US Adults Aged 60 Years or Older
Source: JAMA Netw Open. 2025 May 9;8(5):e258322. doi: 10.1001/jamanetworkopen.2025.8322 (PMC12065041; doi:10.1001/jamanetworkopen.2025.8322)
Supplement: Supplement 2. — Data Sharing Statement [file jamanetwopen-e258322-s002.pdf]

Data Sharing Statement

Fry. Effectiveness and Safety of Respiratory Syncytial Virus Vaccine for US Adults Aged 60 Years or Older. *JAMA Netw Open*. Published May 09, 2025.
doi:10.1001/jamanetworkopen.2025.8322

Data
Data available: No

Additional Information
Explanation for why data not available: The data are not owned by the authors but by Epic Cosmos, a data platform updated daily. Therefore, the authors cannot share the data. Data on Cosmos are available to individuals at institutions that use the Epic platform and contribute to Cosmos. The authors provide full explanations of the methodology so methods can be replicated by others on the Current Cosmos data platform but cannot provide patient data
